# Assessing cardiac mechanics through left ventricular haemodynamic forces

**DOI:** 10.1093/ehjimp/qyae077

**Published:** 2024-07-25

**Authors:** Alberto Aimo, Giorgia Panichella, Iacopo Fabiani, Manuel Garofalo, Angela Ilaria Fanizzi, Maddalena Ragagnin, Alessandra Milazzo, Chiara Zocchi, Annamaria Del Franco, Gianni Pedrizzetti, Iacopo Olivotto, Michele Emdin

**Affiliations:** Interdisciplinary Center for Health Sciences, Scuola Superiore Sant'Anna, piazza Martiri della Libertà 33, 56127 Pisa, Italy; Cardiology Division, Fondazione Toscana Gabriele Monasterio, via Moruzzi 1, 56124 Pisa, Italy; Department of Experimental and Clinical Medicine, University of Florence, 50121 Florence, Italy; Cardiology Division, Fondazione Toscana Gabriele Monasterio, via Moruzzi 1, 56124 Pisa, Italy; Department of Experimental and Clinical Medicine, University of Florence, 50121 Florence, Italy; Department of Experimental and Clinical Medicine, University of Florence, 50121 Florence, Italy; Department of Experimental and Clinical Medicine, University of Florence, 50121 Florence, Italy; Department of Experimental and Clinical Medicine, University of Florence, 50121 Florence, Italy; Cardiovascular Department, San Donato Hospital, 52100 Arezzo, Italy; Department of Experimental and Clinical Medicine, University of Florence, 50121 Florence, Italy; Department of Engineering and Architecture, University of Trieste, Trieste, Italy; Meyer University Hospital, 50139 Florence, Italy; Interdisciplinary Center for Health Sciences, Scuola Superiore Sant'Anna, piazza Martiri della Libertà 33, 56127 Pisa, Italy; Cardiology Division, Fondazione Toscana Gabriele Monasterio, via Moruzzi 1, 56124 Pisa, Italy

**Keywords:** haemodynamic forces, echocardiography, systolic function, cardiomyopathy

## Abstract

Haemodynamic forces (HDFs), which represent the forces exchanged between blood and surrounding tissues, are critical in regulating the structure and function of the left ventricle (LV). These forces can be assessed on cardiac magnetic resonance or transthoracic echocardiography exams using specialized software, offering a non-invasive alternative for measuring intraventricular pressure gradients. The analysis of HDFs can be a valuable tool in improving our understanding of cardiovascular disease and providing insights beyond traditional diagnostic and therapeutic approaches. For instance, HDF analysis has the potential to identify early signs of adverse remodelling and cardiac dysfunction, which may not be detected by standard imaging methods such as bidimensional or speckle-tracking echocardiography. This review aims to summarize the principles of HDF analysis and to reappraise its possible applications to cardiac disorders.

Forces exchanged between the blood and surrounding tissues are known as haemodynamic forces (HDFs).^1^ HDFs represent the cumulative measure of intraventricular pressure gradients (IVPGs) across the entire volume of left and right ventricles (LV and RV, respectively), providing a spatial–temporal representation of the pressure gradients induced by cyclic interaction between blood and tissue boundaries. Due to the tight correlation between segmental wall mechanics and the dynamics of LV filling and ejection,^2–4^ HDFs can be considered fluid dynamics equivalent to deformation imaging.^5^

HDFs have been found to impact the morphogenesis of embryonic hearts^6,7^ and have been implicated in adverse cardiac remodelling in several conditions.^2–4,8–10^ There is indeed specific evidence^9^ that HDF analysis may insights into cardiac physiology that traditional cardiovascular techniques like LV ejection fraction (LVEF) or deformation techniques (such as speckle-tracking echocardiography, STE) cannot offer.^5^ Additionally, the primary clinical application of HDF analysis lies in the early detection of cardiac dysfunction, before symptoms develop. This proactive approach may allow timely intervention and management of several cardiac disorders.

## Principles of haemodynamic forces (HDFs) assessment

The concept that intracardiac blood flow is propelled by IVPGs has been recognized for nearly a century.^11^ These gradients were demonstrated in animal models through cardiac catheterization in the mid-20^th^ century.^12,13^ For example, open-chest dog experiments examined the IVPGs accounting for mitral valve leaflet motion and blood flow across the valve.^14^ These studies demonstrated that efficient LV contraction generates IVPGs that are pivotal for systolic blood ejection and that LV relaxation modulates the amplitude of IVPGs, thereby aiding in diastolic filling.^14^ In the 1990s, non-invasive assessment of diastolic pressure gradients began using spatiotemporal velocity distribution from M-mode colour Doppler^15,16^ and phase-contrast magnetic resonance imaging (MRI).^17,18^ Nonetheless, echocardiographic particle image velocimetry (PIV) faced limitations due to the requirement for contrast agent infusion and high-quality images.^8^ Lately, three-dimensional (3D) phase-contrast MRI, also known as four-dimensional (4D-) flow MRI, emerged as a highly reproducible method and was validated against external reference standards such as laser PIV and numerical models.^19,20^ However, its adoption is limited due to high costs, time consumption, and low availability.

**Figure 1:**
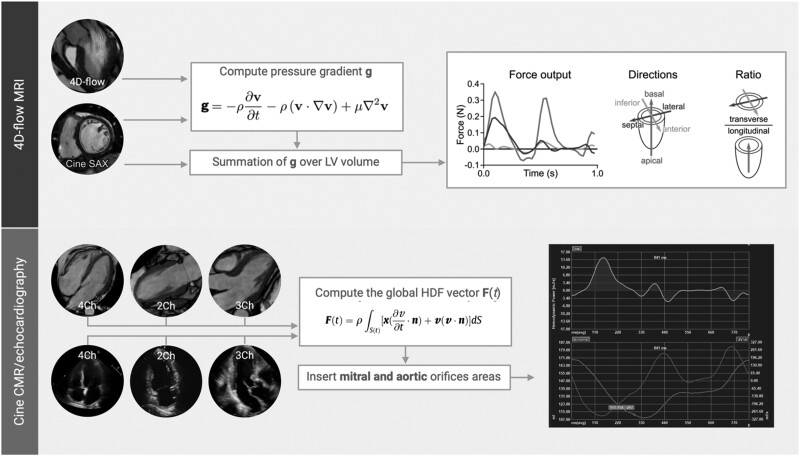
Techniques currently used to assess haemodynamic forces (HDFs). Upper panel: Global haemodynamic forces (HDFs) can be calculated at 4D-flow magnetic resonance imaging (MRI). Left ventricular (LV) endocardial border is manually delineated in cine images. Once the velocity field is known, pressure gradients **g** in the 4D-data set are computed using the Navier–Stokes equation and integrated over the LV ventricular volume to produce the HDF. The equation uses variables such as ***ρ*** for mass density, ***v*** for fluid velocity vector field, ***t*** for time, and ***μ*** for the fluid viscosity. The force vector obtained is decomposed into 3 orthogonal components: basal–apical, lateral–septal, and inferior–anterior. For each component, HDF output, directions, and the transverse/longitudinal ratio are obtained. Lower panel: Thanks to recent advances,^1^ HDFs can be also computer at cine cardiac magnetic resonance (CMR) and echocardiographic images. First, a four, two, and three-chamber view should be acquired. The global HDF vector F(t) is then calculated thanks to the equation here reported. The fluid velocity vector field ***v*(*x, t*)** is measured at fixed points ***x*** at time ***t***, while ***S*(*t*)** represents the closed surface bounding the volume, and ***n*** is the outward unit normal vector. In this way, the force associated with a fluid volume can be evaluated from measurements carried out at the boundaries of the volume regardless of flow phenomena developing inside. This calculation, in fact, requires the velocity over the endocardial boundary (derived from the myocardial movement using the same feature tracking results used for strain and strain rate analysis) and the blood velocity across the valves (which is calculated from the volumetric changes of the LV and the valve area, using the conservation of mass principle). The three-dimensional surface is reconstructed by combining the 3 apical views, and parameters similar to 4D flow are obtained (i.e. haemodynamic force, orientation, and angle ratio). For an explanation of the individual curves shown in the last part of the lower panel, please refer to *Figure 3*.

HDF analysis has recently become more accessible thanks to a mathematical model based on fundamental fluid dynamics and mass conservation principles.^3,4^ This model integrates knowledge of LV geometry, endocardial tissue movement, and the dimensions of the aortic and mitral orifices, by eliminating the need for direct measurements of blood velocities within the LV (*Figure 1*).^1^ As a result, the force exerted by a fluid volume can be determined from images obtained during transthoracic echocardiogram (TTE) or cardiac magnetic resonance (CMR) exams with a post-acquisition data analysis time of approximately 30 min per patient.^21,22^ To facilitate comparisons of HDFs between ventricles of different sizes, LV volume should be normalized.^3^ This includes adjusting for fluid density and gravitational acceleration, which translates a force into a percentage of the static weight of the blood in the LV, and acceleration as a percentage of gravitational acceleration.^5^ By expressing forces and accelerations in dimensionless terms, comparisons between patients can be made, and reference limits can be identified more easily.

## Physiological patterns of HDFs

HDFs in the LV manifest along three primary planes: apical-basal (A-B), lateral–septal (L-S), and anterior-inferior (A-I).^5^ HDFs in the A-B direction are the most consistently reproducible and detectable ones. Analysing HDFs in the RV is more complex due to the variable dominant flow direction during different cardiac cycle phases, which is currently best assessed by CMR.^5^

**Figure 2:**
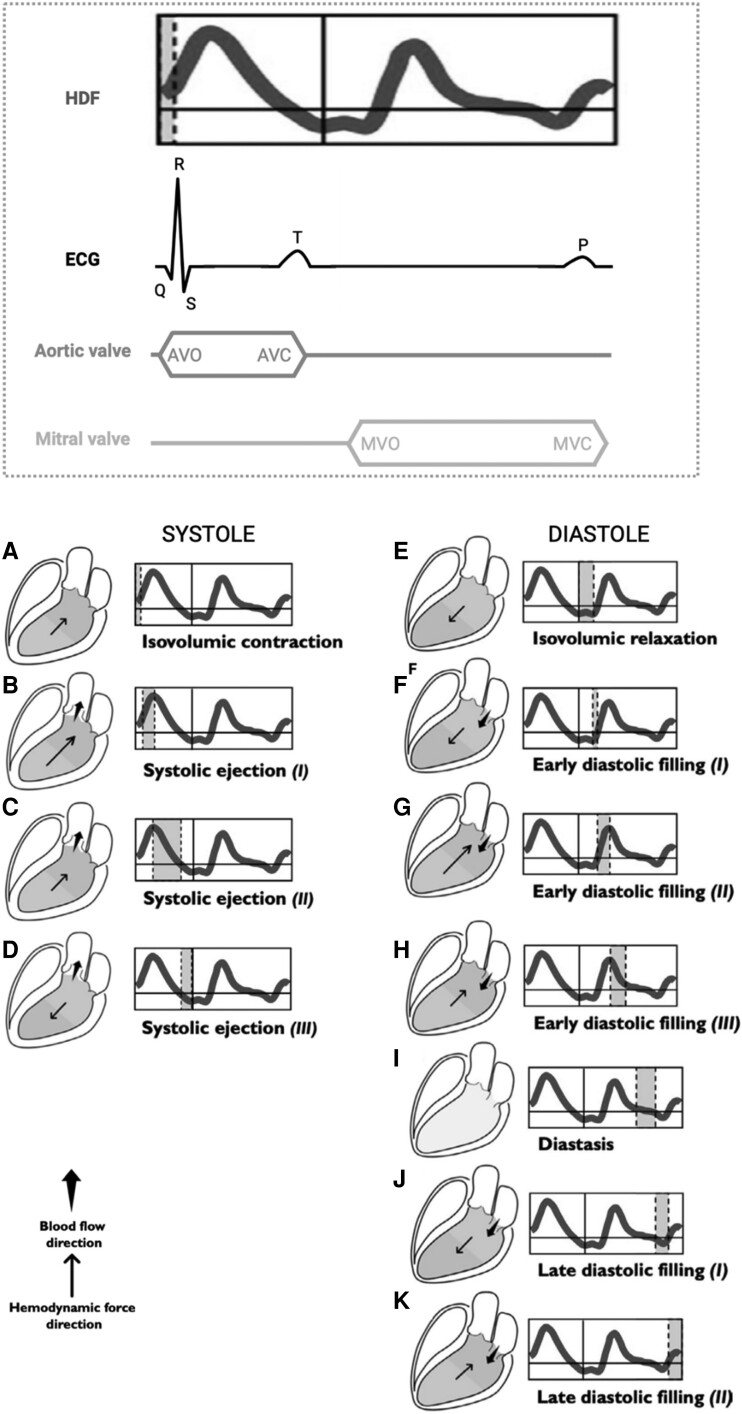
Left ventricular (LV) longitudinal HDFs in systole and diastole. The haemodynamic force (HDF, thin arrow) always flows from the area of higher to the lower pressure area. The absence of intraventricular pressure gradient (IVPG) is displayed in grey. The flow direction (*wide arrow*) goes from the higher to the lower pressure chamber. The start of systole is marked by isovolumic contraction, which occurs between the mitral valve closure (MVC) and the aortic valve opening (AVO), which corresponds to the QRS complex on ECG. During this phase, the longitudinal force shows a positive deflection (*A*) due to an apex-base IVPG, which causes an acceleration of flow towards the base. When the AV opens, systolic ejection begins. Initially, HDFs exhibit a positive ascending phase, because of the increased IVPG (*B*). Once the peak is reached, although ventricular contraction continues, LV starts to lose tension; this causes a decrease in the gradient between the apex and the base in a positive descending phase, as a large amount of blood volume has been ejected (*C*). As systole nears its end, the IVPG reverses (becoming greater at the basal level). At this stage, ventricular flow is decelerating, because aortic pressure exceeds LV pressure and the intraventricular HDF moves in the opposite direction with respect to the flow (*D*) until AV closes (AVC). Then, diastole begins with the isovolumic relaxation (*E*), during which both the AV and MV are closed. During this period, there is no flow between the cardiac chambers. However, because of active myocardial relaxation and recoil of elastic forces generated during the previous systole, the IVPG directed towards the ventricular apex increases, thus generating diastolic suction. When the LV pressure falls below that of the left atrial (LA), MV opens (MVO), and the early diastolic filling begins. Ventricular filling at this stage is passive and the HDF vector continues to be directed towards the LV apex, although the pooling of blood directed towards the apex rapidly resets the HDF (*F*). Subsequently, LV filling continues supported by the upward movement of the mitral plane that displaces the blood contained in the atrium inside the LV. During this phase, the pressure in the LV gradually increases until it exceeds LA pressure, thus inverting the atrioventricular pressure gradient, decelerating the LV filling, and causing HDF to increase in the positive ascending phase (*G*). The reduced flow of blood from the atrium to the LV progressively equalizes the pressures in both chambers, eventually reducing the gradient to zero (*H*). During the diastasis phase, a pressure equilibrium is established between the base and apex (and between LV and LA) (*I*). The occurrence of atrial contraction (P wave on ECG) causes a relative gradient from apex to base, resulting in HDF negative vectors (*J*) and producing the late diastolic filling. As blood accumulates in LV, the ventricular gradient is reversed, and the HDF vector becomes positive (*K*), decelerating the diastolic filling flow, and preparing LV for the systolic ejection phase. *Modified with permission from* Vallelonga *et al*.^5^

HDF analysis offers a better understanding of discrete events during the cardiac cycle, encompassing both systole and diastole (*Figure 2*).

When the HDF vector in the LV moves from the apex towards the base (indicating higher apical than basal pressure), it manifests as a positive deflection above the zero line. Conversely, if basal pressure exceeds apical pressure, it is marked by a negative deflection.^5^ A reduced systolic peak may indicate compromised global myocardial contractility or an abnormal spatial–temporal contractile pattern due to a lack of mechanical or electrical synergy among different segments, leading to reduced force towards the LV outflow tract. The assessment of LV suction dynamics can be based on the magnitude and duration of the negative deflection preceding mitral valve opening. This phase is characterized by changes in LV shape and the presence of a pressure gradient towards the apex linked to the elastic recoiling forces (negative HDFs). Additionally, the pattern of the diastolic portion of the HDF is associated with the load and passive mechanical properties of the LV chamber.^5^

In conclusion, HDF analysis provides a comprehensive graphical representation of the cardiac cycle, integrating the temporal progression of ventricular volumes, LV shape, and intracavitary fluid dynamics.

## HDF parameters

**Figure 3:**
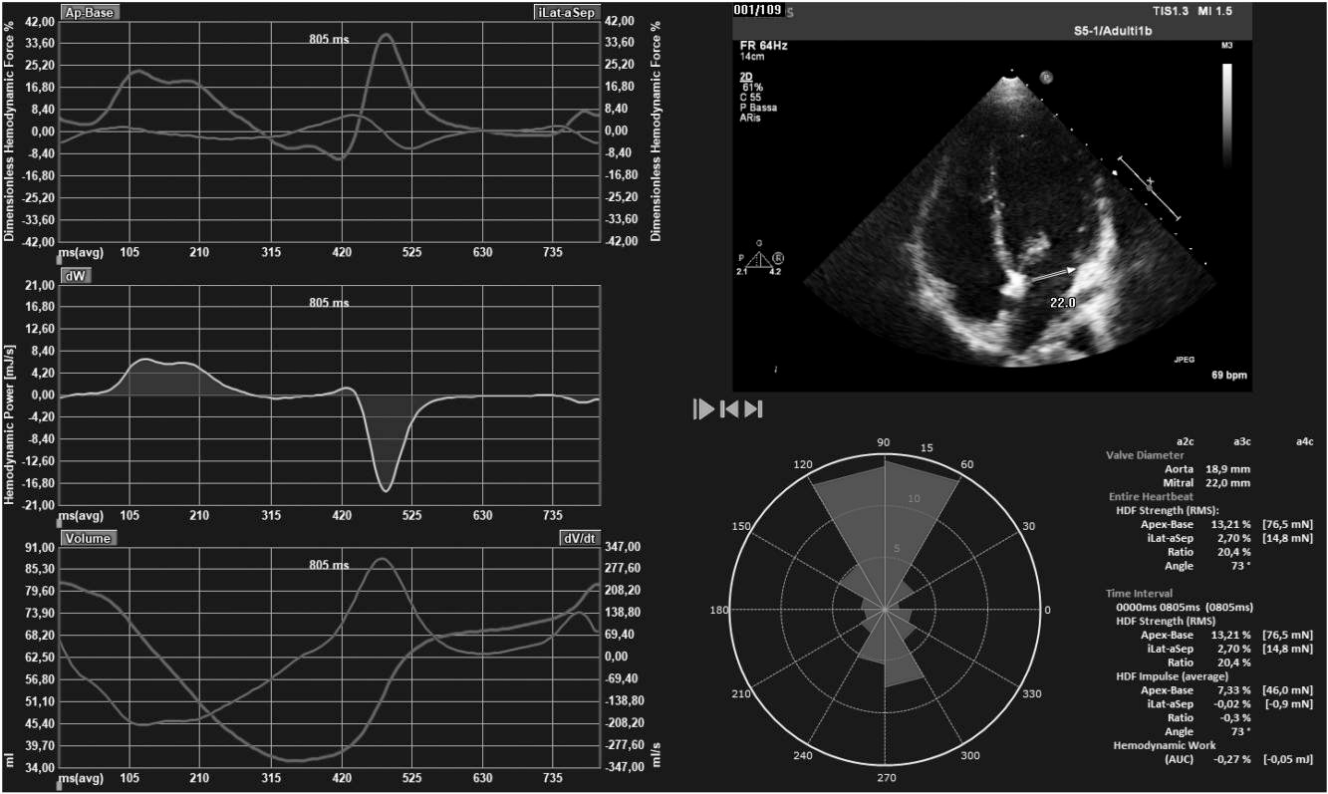
LV longitudinal HDF curves. On the left, from top to bottom: I) Time profile of the left ventricular apical-basal and lateral–septal haemodynamic forces; II) haemodynamic power; III) Time profile of the left ventricular apical-basal and lateral–septal volume curves. At the bottom right, the red isosceles triangles within the polar histogram represent the distribution and intensity of the left ventricular haemodynamic force during the entire cardiac cycle. This latter helps defining the main direction of blood flow during the cardiac cycle. As opposed to the physiological longitudinal orientation of blood flow, pronounced transversal HDF components indicate a deviation from the normal synchrony of segmental myocardial deformation, resulting in abnormally oriented pressure gradients.

Several measures have been proposed to describe LV HDFs. They are mainly based on the time curves displayed in *Figure 3*.

**Figure 4:**
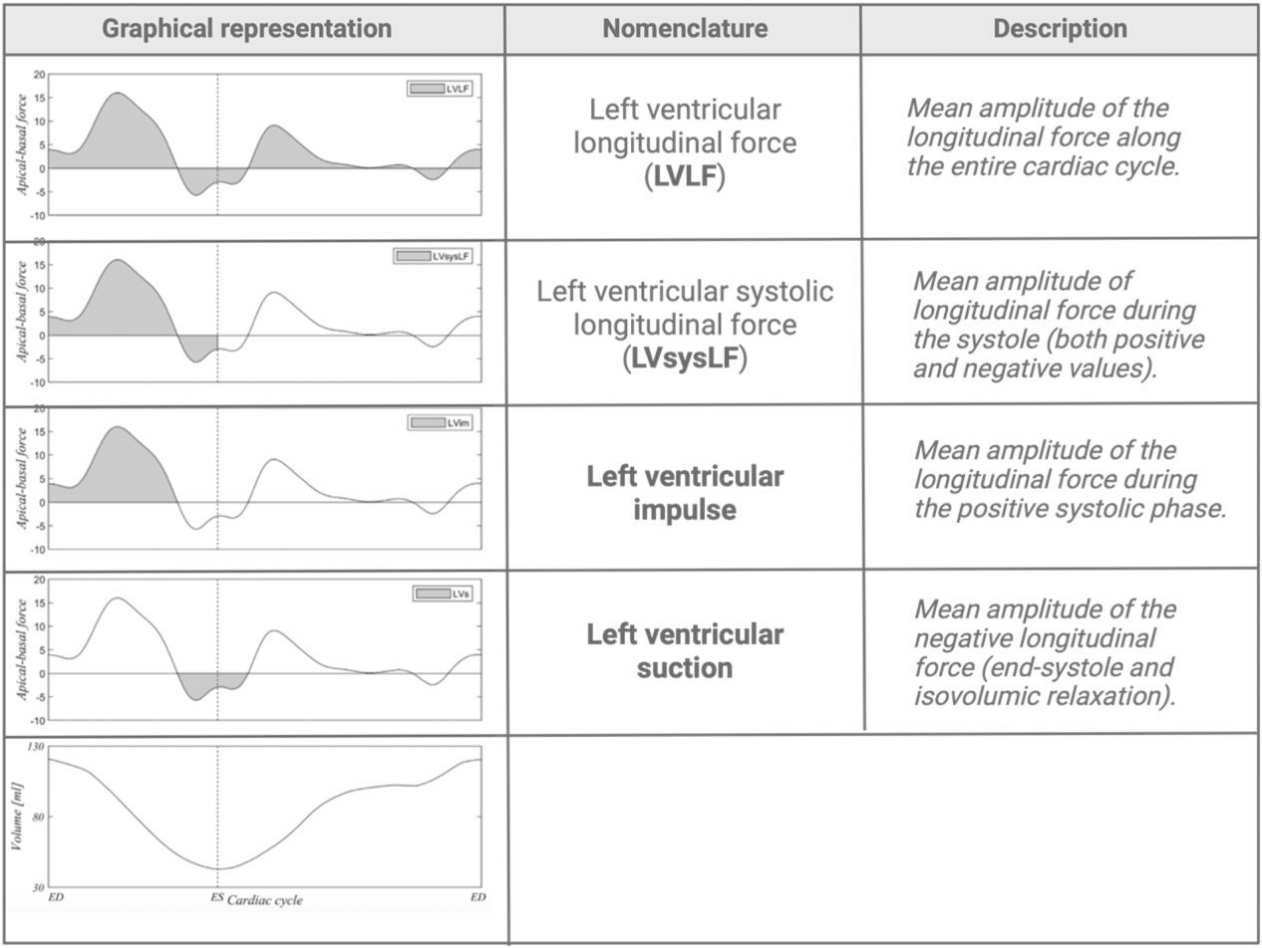
Amplitude intervals commonly used in the calculation of HDFs. *Modified with permission from* Faganello *et al*.^23^

Several parameters can be assessed depending on the features of the specific conditions. HDF parameters can be classified into three main categories:

1. **Amplitude parameters**, which express the mean amplitude of the LV HDFs in different time intervals. These parameters are frequently reported in the literature (*Figure 4*), particularly in the A-B or longitudinal direction. Reference values are reported in *Table 1*.^23^ The *LV longitudinal force* (LVLF) is one of the most commonly reported parameters, representing the mean amplitude of the longitudinal HDF (both positive and negative values) throughout the entire cardiac cycle. LVLF is a surrogate for LV function, which proves to detect structural and functional alterations even earlier than traditional echocardiographic parameters. In patients with heart failure with preserved ejection fraction (HFpEF), significant differences in LVLF have been described as compared to healthy controls, while no differences in LVEF, global longitudinal, or circumferential strain (GLS or GCS, respectively) were detected.^9^
2. **Timing parameters**, which are measures associated with timing of events derived from the HDF curve. Some examples of time intervals include the *duration of LV negative longitudinal force in the transition from systole to diastole* or the *time from the start of relaxation to positive peak of diastolic LV longitudinal force*. These parameters have received limited attention so far.
3. **Orientation parameters**, which are measures associated with the direction of the LV force vector. The most frequently assessed parameter in this category is the *ratio between the transverse force and the longitudinal force.* In literature, this ratio is referred to as the L-S/A-B ratio when applied to TTE, and the short-/long-axis ratio (SAx/LAx) when applied to MRI. This value provides a comparison between longitudinal and transverse components, with the latter representing a form of wasted and inefficient energy. This parameter may help assess LV dyssynchrony^4,24^ or the results of cardiac resynchronization therapy (CRT). Indeed, the L-S/A-B ratio increases in patients with LV dyssynchrony, and its reduction by CRT has important clinical and prognostic implications.^25,26^

**Table 1:** Reference values for left ventricular (LV) longitudinal haemodynamic force (HDF) intervals according to age and gender

	Age 16–39 (*n* = 57 pts)		Age 40–59 (*n* = 64 pts)		Age ≥60 (*n* = 55)	
	Male mean ± SD	Female mean ± SD	Male mean ± SD	Female mean ± SD	Male mean ± SD	Female mean ± SD
LVLF (%)	16.6 ± 4.3	13.5 ± 3.6	17.1 ± 6.7	14.6 ± 3.8	14.3 ± 3.8	12 ± 3.2
LVsysLF (%)	27.9 ± 6.6	19.3 ± 5.1	25.4 ± 9	21.8 ± 6.1	20.1 ± 5.6	17.7 ± 5
LVim (%)	23.9 ± 5.6	16.5 ± 4.8	20.1 ± 8	18.8 ± 5.9	16.1 ± 4.5	15.2 ± 4.5
LVs (%)	8.3 ± 2.1	8.6 ± 2.4	9 ± 2.6	7.7 ± 2.6	9.3 ± 2.3	8.1 ± 2.3

LVLF, left ventricular longitudinal force; LVsysLF, left ventricular systolic longitudinal force; LVim, left ventricular impulse; LVs, left ventricular suction; pts, patients; SD, standard deviation. *Reproduced with permission from* Faganello *et al*.^23^

## HDF analysis: practical considerations

HDF analysis has been most commonly performed on echocardiographic exams. The echocardiographic evaluation of HDF, having a valid dataset available for 2D speckle-tracking echocardiography (STE) analysis, and the dedicated software application (manual calculation of the diameter of the mitral and aortic annulus, mm). HDFs are obtained by offline analysis of echocardiographic DICOM files with dedicated software (QStrain Echo, Medis Medical Imaging, Leiden, the Netherlands). First, the software performs STE analysis of LV in the three routinely acquired apical scans: four-chamber, two-chamber, and three-chamber views. Then, HDFs can be detected through endocardial velocities, LV geometry, and aortic and mitral orifices areas, obtained after measuring the internal diameter of the valve annulus in parasternal long-axis view (mm). Tissue velocities are derived directly from speckle-tracking dataset. The longitudinal component of the HDFs (i.e. basal–apical direction) is the most widely reproducible and detectable force. The instantaneous value of HDFs is normalised by the corresponding value of LV volume to compare patients with different LV sizes. It is then divided by blood density and gravity acceleration, obtaining a dimensionless value corresponding to the force expressed as a percentage of gravity acceleration.

The analysis, which provides quantitative data, can be performed in post-processing with relative time consumption compared to a conventional 2D-strain assessment, with good reproducibility and relatively low costs (software) in any clinical context, albeit with limitations related to highly variable/irregular rhythms and the presence of significant mitral regurgitation. The available evidence in the literature needs to create precise reference values that make the method usable and easily interpretable in routine clinical practice.

As for CMR exams, conventional balanced steady-state free precession images in standard short- and long-axis projections with retrospective ECG gating are also needed to define the LV cavity for subsequent quantification of haemodynamic forces with dedicated software.^22^ Intraventricular pressure gradients are then computed using the Navier–Stokes equations and integrated over the entire LV cavity. The atrioventricular plane is set as the spatial reference system: the apex-base direction is set as perpendicular to the atrioventricular plane, the lateral wall-septum direction is set as perpendicular to the apex-base direction and aligned to the LV outflow tract, and the inferior–anterior direction is set as perpendicular to both the apex-base and the lateral wall-septum directions.

## HDF reference values

Reference values and the impact of demographic and technical factors on HDFs are still being investigated. In a study by Faganello *et al*., 176 subjects (age range, 16–82 years old; 51% women), with no cardiovascular risk factors or any relevant diseases, were analysed.^23^ LVLF, LVsysLF, and LV impulse were all higher in men than women (16.2 ± 5.3 vs. 13.2 ± 3.6; 25.1 ± 7.9 vs. 19.4 ± 5.6, and 20.4 ± 7 vs. 16.6 ± 5.2, *P* < 0.0001, respectively). A statistically significant decline with age (*P* < 0.001) was noticed for these parameters.^23^ Reference values according to age and gender are shown in *Table 1*.

**Figure 5:**
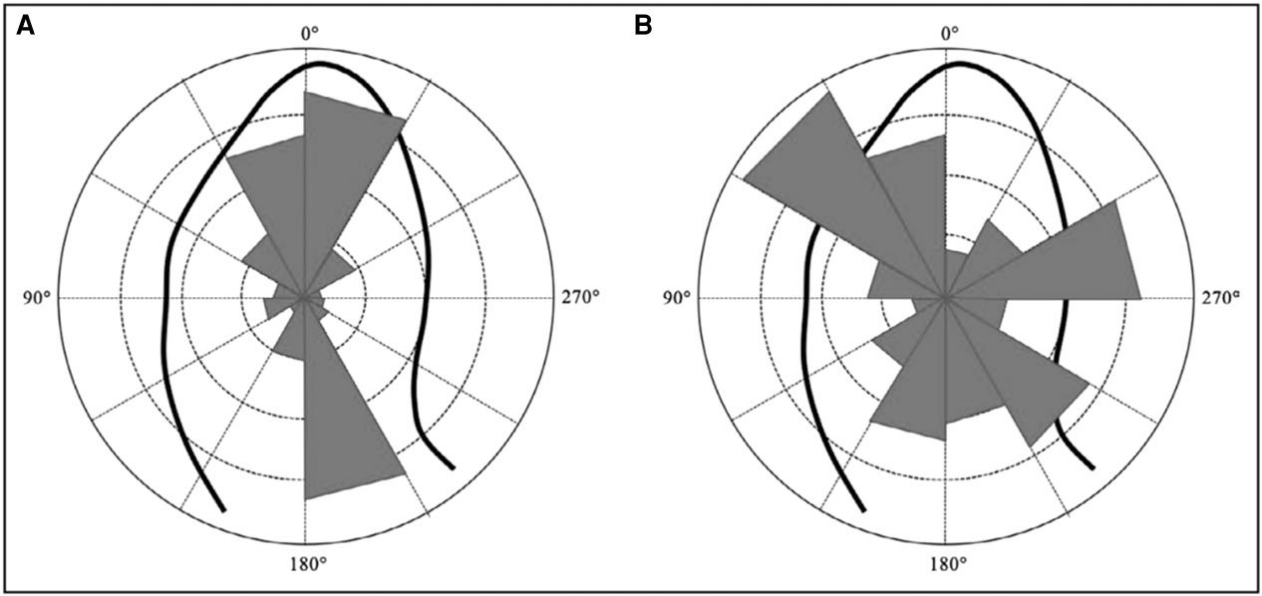
Intensity-weighted polar histogram of HDFs orientation. The distribution and intensity of the left ventricular haemodynamic force during the entire cardiac cycle are shown by red isosceles triangles within a polar histogram. *A*, physiological flow orientation with a mainly longitudinal (apex-base) directed forces; *B*, pathological flow orientation with a prevalent transversal (septal-lateral or inferior-anterior) directed forces. *Reprinted with permission from* Vallelonga *et al.*^5^

Typically, the main HDFs in the heart are oriented predominantly along the longitudinal or A-B direction, as shown in *Figure 5A*. This orientation optimizes the energy required by the heart to generate stroke volume. It coincides with the natural direction of myocardial deformation, aiding blood flow from the base to the apex during diastole and in the reverse direction during systole. However, due to the three-dimensional structure of the heart, a slight transverse (side-to-side) component of HDFs is an integral part of the heart’s complex dynamics.

Pronounced transversal HDF components indicate a deviation from the normal synchrony of segmental myocardial deformation, resulting in abnormally oriented pressure gradients (*Figure 5B*). When a transversal HDF component has a comparable amplitude to the longitudinal component, this lateral force does not effectively contribute to the cardiac pumping or filling activities, unlike the productive longitudinal forces. Significant transversal forces may indicate underlying cardiac disorders and should be properly investigated. In patients with established cardiac disease, HDF analysis may capture a redirection of haemodynamic forces towards the longitudinal direction, which predicts response to treatment and possibly reverse remodelling.

Irregularities at the blood-wall interface may alter HDF patterns by disrupting IVPGs, leading to flow diversion and changes in vorticity patterns. These changes culminate in a reduction in endocardial shear stress, which contributes to adverse ventricular remodelling.^5^

## HDF analysis: clinical applications

HDF analysis can be applied to many clinical settings, thanks to its versatility and easy applicability (*Table 2*).

### Heart failure

**Figure 6:**
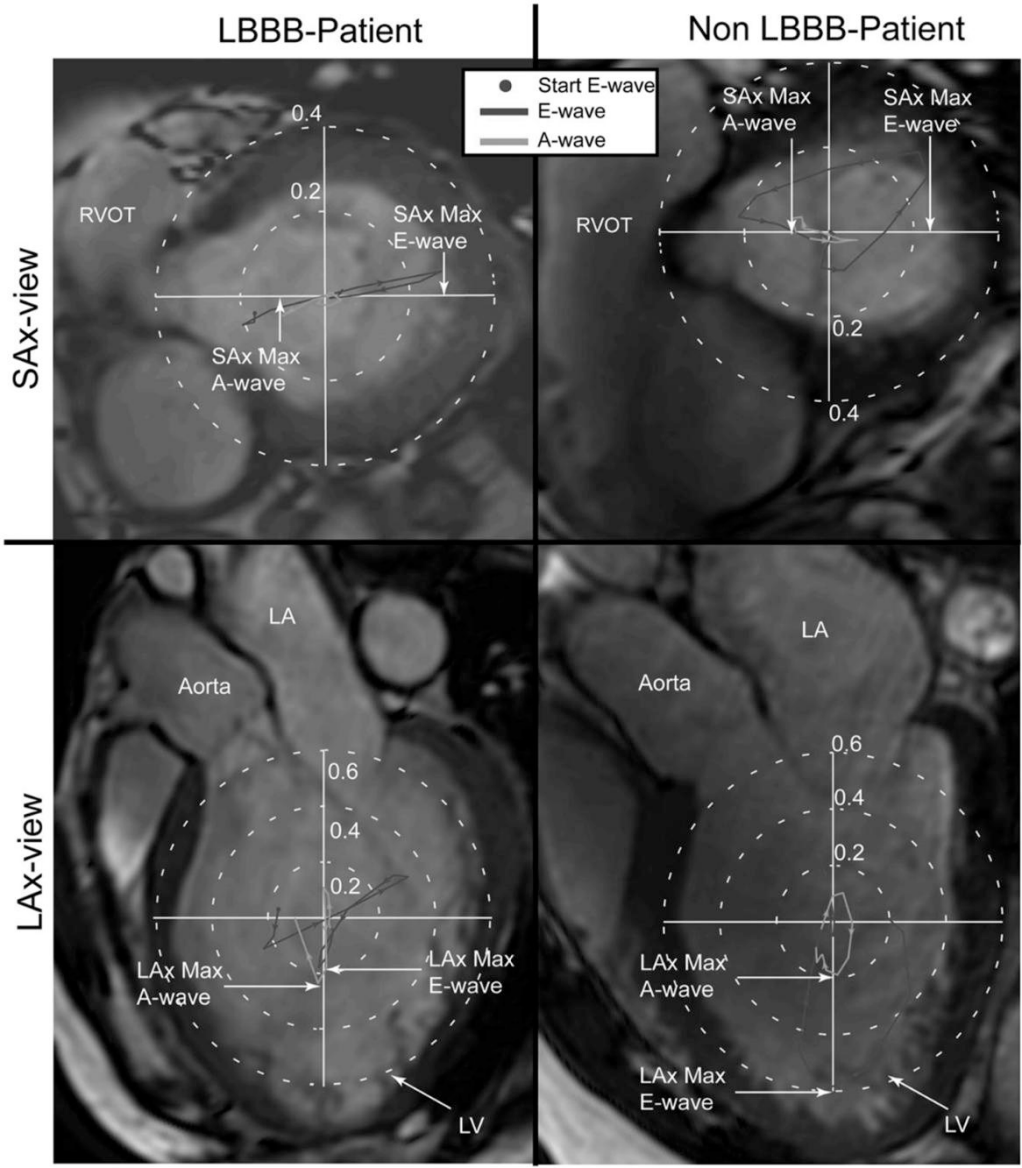
HDFs in a patient with left bundle branch block (LBBB) in comparison with a second patient without LBBB. The figure illustrates the HDF plots in a patient with LBBB (*left*) and in a patient withoutLBBB (*right*), projected onto a short-axis (SAx) image (*top*) and long-axis (LAx) image (*bottom*). The HDF plots are colour-coded according to diastolic phases, E-wave and A-wave. The ‘SAx_max_/LAx_max_ force’ ratio was defined as the ratio between the maximum force along the anteroseptal-to-inferolateral axis in the SAx-view and the maximum force along the apex-to-base axis in the LAx three-chamber view. E-wave, early diastolic filling; A-wave, late diastolic filling; LA, left atrium; LV, left ventricle. *Reprinted with permission from* Eriksson *et al.*^4^

HDFs have been proposed as a useful tool to early detect alterations in cardiac function and predict disease outcome in HF. Erikkson *et al*. sought to investigate the impact of left bundle branch block (LBBB)-related dyssynchronous LV relaxation on global LV diastolic haemodynamics and function. 4D-flow MRI data were successfully acquired in the presence or not of LBBB for HF patients matched by age, gender, heart rate, and LV characteristics. The SAx_max_/LAx_max_-ratio, a measure of the deviation of the LV HDFs from the main flow direction, resulted to be higher during the early diastolic filling phase in the patients with LBBB compared to those without (0.90 vs. 0.62; *P* = 0.054), while no intergroup difference was observed during late diastolic filling [0.33 vs. 0.27; *P* = 0.38 (*Figure 6*)].^4^ Similar results were confirmed by, Arvidsson *et al*.^24^ The SAx/LAx ratio was higher in both systole and diastole compared to controls, indicating a reduced longitudinal alignment of forces in patients. In some cases, a significant fraction of forces was observed in the I-A direction. Furthermore, the systolic force ratio was significantly associated with global longitudinal peak systolic strain (GLPSS, i.e. the average of the peak systolic strain for each of the long-axis views) and LVEF, suggesting that decreased longitudinal function is coupled to low longitudinal forces.^24^

Changes in HDFs may reflect early alterations in cardiac function that can be of added value for the early diagnosis of HFpEF.^27^ Lapinskas *et al*. analysed HDFs assessed using CMR images from patients with HFpEF, mildly reduced (HFmrEF), and reduced ejection fraction (HFrEF).^9^ There was a direct correlation between LVLF and LVEF (*r* = 0.71; *P* < 0.001). Interestingly, LVLF was significantly decreased in patients with HFpEF compared to healthy controls (0.169 ± 0.036 vs. 0.243 ± 0.056; *P* = 0.008), whereas no differences were observed in terms of LVEF (*P* = 0.347), GLS (*P* = 0.875) or GCS (*P* = 0.433). These results may open new perspectives for HFpEF phenotyping and early detection of HF.^9^ A similar study did not reveal any differences in HDF parameters between HFpEF patients and healthy controls.^28^ However, it is important to note that this study was performed on a small sample size and among patients with a heterogeneous clinical phenotype. Overall, positive results predominate. This is the case of Backhaus *et al*. who evaluated HDFs in 34 patients with HFpEF and 34 with non-cardiac dyspnoea according to pulmonary capillary wedge pressure (PCWP).^29^ Patients with HFpEF had lower LVLF (15.8% vs. 18.3%; *P* = 0.035), systolic peak (39% vs. 52%; *P* = 0.002) and impulse (21% vs. 25%; *P* = 0.006) forces as well as lower diastolic deceleration (9.1% vs. 7.1%; *P* = 0.044) and late diastolic filling (−3.8% vs. −5.4%; *P* = 0.029) compared to non-cardiac dyspnoea.^29^ Impaired systolic peak was associated with cardiovascular mortality and hospitalization [hazard ratio (HR) 0.95; *P* = 0.016], and was superior to LV GLS assessment in predicting outcomes [area under the curve (AUC) 0.76 vs. 0.61; *P* = 0.048].^29^

The gold standard for the assessment of diastolic function is the detection of increased LV filling pressures (ILFP) at right heart catheterization (RHC). Recently, a retrospective study evaluated the application of HDFs in such a setting.^30^ Of 67 patients, 33 (49%) showed ILFP at RHC. Diastolic longitudinal force (DLF), a new parameter showing the mean amplitude of LVLF during diastole, was associated with the presence of ILFP [odds ratio (OR) 0.84; *P* = 0.046].^30^ A scoring system including DLF, LVEF, left atrium (LA) enlargement, and e’ septal showed an AUC of 0.83. Interestingly, the score showed a sensitivity of 67% and a specificity of 94% when applied to patients classified as having ‘indeterminate diastolic function’ according to the current recommendations.^30^

**Figure 7:**
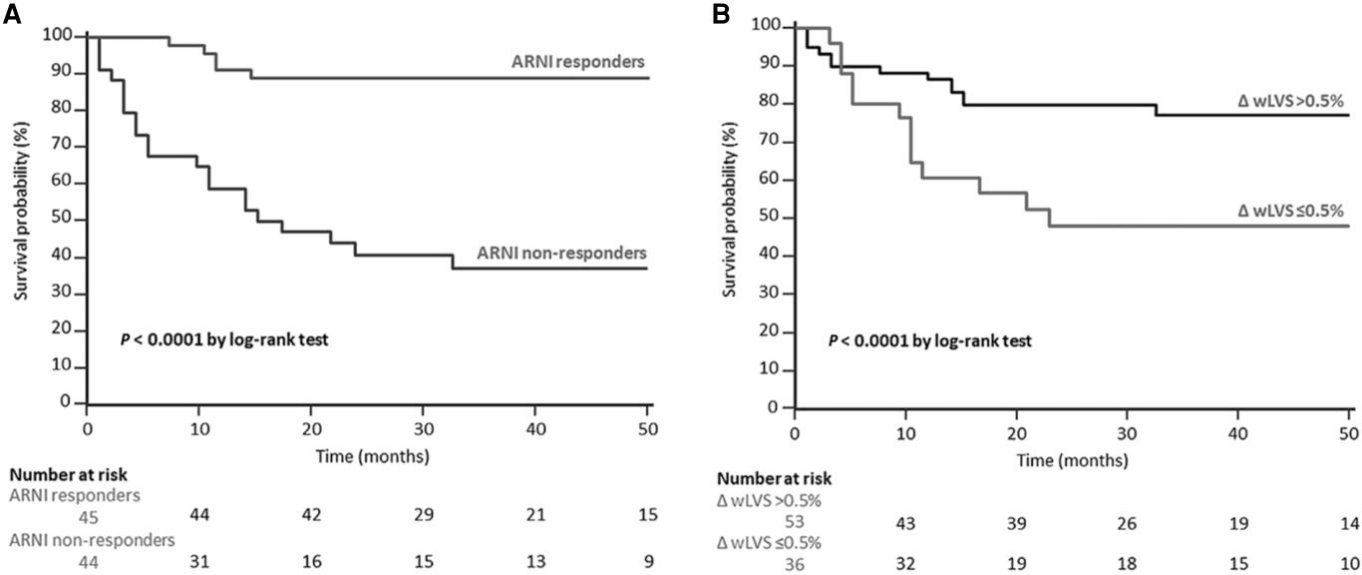
Survival analyses in patients on sacubitril/valsartan. Kaplan–Meier survival analysis for the composite endpoint (all-cause death, hospitalization due to worsening heart failure, and new-onset atrial fibrillation) after a median follow-up of 32.5 months. The patients are stratified by angiotensin receptor-neprilysin inhibitor (ARNI) responders and non-responders (*A*) and according to Δwhole cardiac cycle left ventricular strength (ΔwLVS > 0.5% vs. ΔwLVS > 0.5%) (*B*). Δ indicates the difference measured between the 6-month ARNI-response protocol and baseline evaluation. *Reprinted with permission from* Fabiani *et al*.^32^

HDF analysis can also help predict therapeutic response and the risk of adverse cardiovascular outcomes in patients with HF. For example, sacubitril/valsartan induces reverse remodelling with increased myocardial contractility and improved HDF distribution.^31^ After 6 months of therapy, LV indexed volumes decreased, LVEF and GLS improved, and re-alignment of HDFs occurred, with a reduction indiastolic L-S/A-B HDF ratio (23% vs. 20%; *P* < 0.001).^31^ In a recent study, we evaluated the predictive value of HDFs on 6-month treatment response to sacubitril/valsartan in 89 HFrEF patients.^32^ Patients were categorized as responders if not experiencing any adverse event and exhibiting a reduction of at least 50% in N-terminal pro–B-type natriuretic peptide (NT-proBNP) and/or an increase of 10% or more in LVEF over 6 months. Only LVLF differed between responders and not responsers, and was higher in responders (4.4% vs. 3.6%; *P* = 0.01). LVLF was the only independent predictor of sacubitril/valsartan response at multivariable logistic regression analysis [OR 1.36; 95% confidence interval (CI) 1.10–1.67].^32^ During a 33-month median follow-up, an increase in LVLF of 0.5% at 6 months was an independent predictor of the composite endpoint of HF-related hospitalization, atrial fibrillation (AF), and cardiovascular death (HR, 0.76; *P* < 0.001), after adjusting for clinical and instrumental variables (*Figure 7*).^32^

### Cardiac resyncronization therapy

The order of electrical activation can affect the orientation of blood flow.^8^ This relationship was first demonstrated by Pedrizzetti *et al*. in 2016.^8^ Thirty HF patients underwent echocardiography before CRT implantation, at follow-up after more than 6 months, and after temporary CRT discontinuation (<5 min). LV mechanics were investigated through speckle-tracking imaging, and intraventricular fluid dynamics through echographic PIV. Patients with active CRT presented a distribution of HDFs predominantly aligned along the A-B direction; when the CRT was discontinued, the flow momentum deviated by developing components along the transversal directions. Moreover, the deviation of flow momentum highly correlated with the degree of volumetric reduction after CRT.^8^ HDFs may therefore have a potential impact on patient selection for CRT and pacing optimization. This was further explored in a retrospective study.^25^ Thirty-eight patients underwent echocardiographic assessment of HDFs before and after CRT. After a follow-up of ≥6 months, 71% of patients were classified as responders [reduction of LV end-systolic volume indexed (LVESVi) of at least 15%].^25^ No significant changes were observed after the CRT implant in terms of LVEF and strain metrics. Conversely, a significant reduction of the L-S/A-B ratio was found (0.46 vs. 0.39; *P* = 0.011). This variation strongly correlated with LVESVi change at follow-up. Additionally, the ratio showed good accuracy in predicting response to CRT (AUC 0.891; *P* < 0.001), with an optimal cut-off of −15.1% (sensitivity and specificity of 73% and 92%, respectively).^25^

Laenens *et al*. investigated the role of HDFs in 197 patients with LVEF ≤35%, QRS duration ≥130 ms, and LBBB.^26^ As expected, LVLF was significantly worse in patients with HF vs. healthy controls throughout the entire heart cycle in terms of both amplitude (4.8% vs. 9.5%; *P* < 0.001) and orientation (66.2 vs. 71.8; *P* < 0.001). Focusing on LV impulse, longitudinal HDFs were still weaker (4.8% vs. 10.1%; *P* < 0.001) and had a worse orientation (74° vs. 77°; *P* < 0.001) in HF patients. Immediately after CRT implantation, HF patients showed an amelioration of LV impulse (4.8% vs. 5.3%; *P* = 0.002) and systolic force vector angle (74° vs. 75°; *P* = 0.036) confirming the previous hypothesis of an ‘acute’ impact of CRT on HDF; these results were also confirmed at 6-month follow-up.

On the opposite view, HDFs may identify patients who are unlikely to benefit from CRT. Pola *et al*. evaluated HDFs at 4D-flow MRI in 22 HF patients with LVEF <35% and LBBB at baseline and 6 months after CRT. Non-responders (i.e. LVESVi reduction, <15%) had smaller DLF (0.09 vs. 0.1; *P* = 0.047) and higher diastolic L-S/A-B ratio (0.89 vs. 0.67; *P* = 0.004) compared to responders. A diastolic L-S/A-B ratio >0.87 identified CRT non-responders with 57% sensitivity and 100% specificity (AUC, 0.88; *P* = 0.005).^33^ Non-responders had smaller systolic HDF in the I-A direction, but no differences were found in other directions.

In summary, the HDF analysis provides valuable insight into the magnitude and orientation of IVPGs in patients with HF undergoing CRT. The evaluation of the magnitude and orientation of HDFs in CRT recipients may represent a novel promising tool for determining CRT candidacy, optimizing parameters, and evaluating effectiveness.

**Table 2:** Summary of the studies evaluating HDFs in clinical settings

Application	Population	Technique	Results	References
HF	HF with LBBB (*n* = 9) HF without LBBB (*n* = 9)	4D-flow MRI	HF with LBBB: more transversal forces during early but not late diastole The greater the conduction abnormality, the greater the discordance of HDF orientation	Eriksson *et al*. ^4^
HF	HF and LV dyssynchrony (*n* = 31) Control subjects (*n* = 39)	4D-flow MRI	Patients have higher Sax/LAx ratio	Arvidsson *et al*. ^24^
HF	HFpEF (*n* = 12) HFmrEF (*n* = 12) HFrEF (*n* = 12) Healthy volunteers (*n* = 12)	Cine CMR	Positive correlation between LVEF and LVLF HFpEF: lower LVLF, no differences in LVEF, GLS, or GCS	Lapinskas *et al*. ^9^
HF	HF pts undergoing RHC (*n* = 67)	TTE	DLF was associated with the presence of increased LV filling pressure. A multiparametric score including DLF successfully reclassify patients with currently undetermined diastolic function	Airale *et al*. ^30^
HF	Pts with HFrEF (*n* = 50) treated with ARNI	TTE	After 6 months, ARNI treatment is associated with decreased LV volumes, increased LVEF and GLS, and improved alignment of HDFs	Monosilio *et al*. ^31^
HF	Pts with HFrEF (*n* = 89) treated with ARNI	TTE	LVLF is the only predictor of ARNI response at 6 months LVLF is an independent predictor of MACE	Fabiani *et al*.^32^
HFpEF	Pts with HFpEF (*n* = 34) and pts with non-cardiac dyspnoea (*n* = 34)	Cine CMR	HFpEF had lower LVLF, systolic peak, LVsysLF, LV impulse, as well as lower diastolic deceleration and atrial thrust than pts with non-cardiac dyspnoea Impaired systolic peak was superior to GLS in predicting CV mortality and hospitalization	Backhaus *et al*.^29^
HFpEF	HFpEF (*n* = 16), HFmrEF (*n* = 9), HFrEF (*n* = 16) pts and healthy controls (*n* = 34)	4D-flow MRI	Positive correlation between LVEF and LVLF No differences between HFpEF pts and healthy controls in HDFs, whether indexed to LV volumes or not	Arvidsson *et al*. ^28^
CRT	CRT pts (*n* = 30) pre and post CRT	Echo-PIV	Discontinuation of CRT → deviation of blood flow momentum in the transversal direction This deviation correlates with the degree of volumetric reduction after CRT	Pedrizzetti *et al*.^8^
CRT	CRT pts (*n* = 38) pre and post CRT	TTE	L-S/A-B ratio decreases at 6 months after CRT implant, with no changes in LVEF or strain measures The ratio variation correlates with LVESVi variation A cut-off of −15.1% in δ(L-S/A-B ratio) accurately predicts response to CRT	Dal Ferro *et al*.^25^
CRT	Pts (*n* = 197) with LVEF ≤35%, QRS duration ≥130 ms and LBBB at baseline and 6 months after CRT (−ON and -OFF)	TTE	Lower magnitude and worse orientation of HDFs in HF pts vs. healthy controls LVLF amplitude and angle, and L-S/A-B ratio improved after CRT When CRT was deactivated, LVLF remained unchanged while the LVsysLF angle worsened significantly	Laenens *et al*.^26^
CRT	Pts (*n* = 22) with LVEF ≤35% and LBBB at baseline and 6 months after CRT	4D-flow MRI	Non-responders to CRT have lower diastolic HDFs and higher L-S/A-B ratio A cut-off of 0.87 in L-S/A-B ratio accurately predicts response to CRT	Pola *et al*. ^33^
DCM	DCM patients (*n* = 10) with mild–moderate ventricular disfunction and healthy controls (*n* = 10)	4D-flow MRI	Longitudinal HFDs are redirected towards the transversal direction in DCM patients	Eriksson *et al*.^34^
DCM	DCM pts (*n* = 447)	Cine CMR	A temporary HDF reversal during systolic–diastolic transition is present in 33% pts Such reversal is associated with MACE	Vos *et al.*. ^21^
AS	Pts undergoing TAVI (*n* = 25)	TTE	LVLF, LVsysLF, LV impulse, and orientation parameters significantly improved after TAVI No changes in LVEF or GLS	Vairo *et al*. ^35^
AS	Pts (*n* = 253) with preserved LVEF and mild (*n* = 87), moderate (*n* = 77), and severe (*n* = 89) AS	TTE	STE and HDF parameters declined as the AS became severe LVsysLF was the only parameter independently associated with AVR and all causes mortality	Faganello *et al*.^36^
MI	STEMI pts (*n* = 49) at baseline and after 4 months Healthy controls (*n* = 21)	CMR	Higher diastolic L-S/A-B ratio is an independent predictor of adverse remodelling at 4 months	Filomena *et al*.^37^
PH	Pts with pPH (*n* = 31) and healthy controls (*n* = 22)	Cine CMR	No differences in systolic function (LVsysLF) Pts had lower LA reservoir and conduit strain, impaired diastolic suction, and lower E-deceleration forces	Vos *et al*.^38^
PH	Pts with pPH (*n* = 20) and healthy controls (*n* = 12)	4D-flow MRI	Biventricular HDFs were larger in pts than controls in all three directions	Pola *et al*. ^39^
ToF	Pts with rToF (*n* = 18) and PR >20% and healthy controls (*n* = 12)	4D-flow MRI	Pts had less aligned systolic and diastolic LV HDFs and higher RV HDFs Altered HDFs did not normalize after PVR	Sjöberg *et al*. ^10^
ToF	Pts with rToF (*n* = 36)	4D-flow MRI	Ventricular remodelling in rToF is related to HDFs magnitude and direction, global and regional functional parameters, and exercise intolerance	Kollar *et al*. ^40^
ToF	Pts with rToF (*n* = 68) and healthy controls (*n* = 20)	Cine CMR	rToF patients have abnormal diastolic HDF Diastolic HDFs are correlated to PR, RV function, exercise capacity, and RVOT vorticity	Loke *et al*. ^41^

A-B, apical–basal; ARNI, angiotensin receptor-neprilysin inhibitor; AS, aortic stenosis; AVR, aortic valve replacement; CMR, cardiac magnetic resonance; CRT, cardiac resynchronization therapy; DCM, dilated cardiomyopathy; DLF, diastolic longitudinal force; EF, ejection fraction; GCS, global circumferential strain; GLS, global longitudinal strain; HDF, haemodynamic force; HF, heart failure; HFmrEF, HF with mid-range ejection fraction; HFpEF, HF with preserved ejection fraction; HFrEF, HF with reduced ejection fraction; LA, left atrium; L-S, lateral–septal; LBBB, left bundle branch block; LV, left ventricular; LVESV, left ventricular end-systolic volume; LVLF, LV longitudinal force; LVsysLF, LV systolic longitudinal force; LVim, LV impulse; MACE, major adverse cardiovascular events; MRI, magnetic resonance imaging; MV, mitral valve; PIV, particle imaging velocimetry; pPH, precapillary pulmonary hypertension; PR, pulmonary regurgitation; pts, patients; PVR, pulmonary valve replacement; RHC, right heart catheterization; RV, right ventricular; RVOT, RV outflow tract; SAx, short axis; TAVI, transcatheter aortic valve implantation; ToF, tetralogy of Fallot; TTE, transthoracic echocardiography; wLVS, whole cardiac cycle LV strength.

### Dilated cardiomyopathy

Impaired intracardiac flows are a significant contributor to adverse LV remodelling in dilated cardiomyopathy (DCM). In a study by Eriksson *et al*., HDF patterns along both long and short axes were analysed using 4D-flow MRI in 10 patients with DCM and compared to 10 healthy controls.^34^ HDFs in DCM patients were redirected towards the short axis direction. SAx/LAx ratio was significantly larger during diastole at both E- (0.53 vs. 0.19; *P* < 0.0001) and A-wave (0.52 vs. 0.32; *P* < 0.03) in the DCM group compared to normals.^34^ This suggests that, similarly to HF, early adverse cardiac remodelling can be manifested as a negative remodulation of forces from the longitudinal axis towards the transversal direction. This results in a wasted effort by the LV as it does not effectively contribute to the ejection or filling processes.

Different HDF patterns may also provide thoughtful information. In a study by Vos *et al*., 168 out of 447 DCM patients (33%) showed a temporary pressure gradient reversal in systolic–diastolic transition, which hindered diastolic filling.^21^ Such pressure reversal was found to predict worse outcomes in terms of HF hospitalizations, life-threatening arrhythmias, and sudden cardiac death (HR, 2.57; *P* = 0.047). In cases where pressure reversal was absent, lower LVLF, LVsysLF, and E-wave decelerative force proved to be powerful independent predictors of the same outcome.^21^

### Aortic stenosis

Recent studies have shown that HDFs are more effective than traditional echocardiographic parameters in detecting subtle myocardial dysfunction in aortic stenosis (AS). In a study of 253 patients with AS and preserved LVEF, LVsysLF was the only parameter, among HDF and STE ones, that was independently associated with aortic valve replacement and all-cause mortality on multivariable Cox regression analysis (HR, 0.94; 95% CI, 0.89–0.99; *P* = 0.012).^36^

HDF analysis was also conducted in 25 patients with severe AS before and after transcatheter aortic valve replacement (TAVR).^35^ Post-TAVR evaluation was performed 2.4 ± 1 days after the procedure. HDF amplitude parameters all significantly improved after the procedure: LVLF [mean difference (MD) 1.79%, *P* < 0.001], LVsysLF (MD, 2.6%; *P* < 0.001), and LV impulse (MD, 2.9%; *P* < 0.001). Similarly, the HDF orientation parameters improved, as represented by LVLF angle (MD, 1.5°; *P* = 0.041) and LV impulse angle (MD, 2.16°; *P* = 0.004).^35^ In contrast, GLS and LVEF did not show any significant differences before and after the procedure. Echocardiographic analysis of HDFs could therefore help to differentiate patients with early LV function recovery after TAVR.

### Myocardial infarction

Altered HDFs are thought to play a crucial role in adverse remodelling after myocardial infarction. In a study conducted on 49 reperfused ST-segment elevation myocardial infarction (STEMI) patients, HDFs were computed at CMR at baseline (1 week after STEMI) and at follow-up (4 months). LV adverse remodelling was defined as a relative increase in LVESVi of at least 15% compared with the baseline. At baseline, STEMI patients had worse HDFs in terms of both amplitude and orientation as computed during the entire heartbeat, systole, and diastole. At univariate logistic regression analysis, larger infarct areas, higher L-S/A-B ratio, and lower transversal forces amplitude were associated with adverse remodelling at follow-up. In the multivariable logistic regression analysis, only higher diastolic L-S/A-B ratio remained an independent predictor of adverse remodelling (OR, 1.1; *P* = 0.04).^37^

### Pulmonary hypertension

Precapillary pulmonary hypertension (pPH) is a condition where understanding the underlying pathophysiological mechanisms is crucial to guide and improve treatment. As LVEF is typically preserved in pPH, it is important to assess LV dysfunction using more sensitive techniques such as HDFs. Compared to healthy volunteers, pPH patients showed to have a worse diastolic function in terms of impaired diastolic suction (−9.1 vs. −6.4; *P* = 0.02) and E-wave deceleration force (8.9 vs. 5.7; *P* < 0.001).^38^ Moreover, a recent study examined biventricular HDFs at 4D-flow MRI in 20 pPH patients and 12 matched controls.^39^ Biventricular HDFs resulted larger in patients than controls in all three directions. In the RV, in particular, patients exhibited higher systolic diaphragm-outflow tract (2.1 vs. 1.4; *P* = 0.003) and septum-free wall HDFs (0.64 vs. 0.42; *P* = 0.007), as well as higher diastolic A-B (1.4 vs. 0.87; *P* < 0.0001), diaphragm-outflow tract (0.80 vs. 0.47,;*P* = 0.005), and septum-free wall HDFs (0.60 vs. 0.38; *P* = 0.003). Altogether, these results suggest that the RV compensates for the increased afterload in part by augmenting transverse forces, while LV haemodynamic abnormalities are mainly a result of underfilling rather than intrinsic ventricular dysfunction.^39^

### Tetralogy of Fallot

Patients who have undergone repair for tetralogy of Fallot (rToF) might develop chronic pulmonary regurgitation (PR) with progressive RV dysfunction and decreased exercise capacity. Currently, progressive RV dilatation and RV systolic dysfunction are the main indicators for pulmonary valve replacement (PVR); however, the optimal timing remains controversial, making a better understanding of rToF pathophysiology of the essence for guiding the clinical management of these patients. Biventricular HDFs were quantified for the first time in a study by Sjöberg *et al*. on 18 patients with rToF and pulmonary regurgitation >20%.^10^ LV HDFs were less aligned to the main blood flow direction, whereas RV HDFs were higher along the pulmonary regurgitant and tricuspid inflow directions in rToF patients compared with control subjects.^10^ Differences in HDFs vs. control subjects remained even after PVR, suggesting that biventricular pumping does not return to normal after surgery.^10^ Similar results were confirmed in a study by Loke *et al*., which additionally showed that pulmonary regurgitation (%) in rToF correlates with diaphragm-to-RV outflow tract (RVOT) HDF amplitude and impulse (*r* = 0.578, *P* < 0.0001 and *r* = 0.508, *P* < 0.0001, respectively).^41^ Diastolic RV HDFs were also found to be correlated with RV function, exercise capacity (VO_2-max_), and RVOT vorticity.^41^ Finally, Kollar *et al*. combined for the first time statistical shape modelling with 4D-flow data to explain the interplay between RV shape, HDFs, and clinical dysfunction in rToF.^40^ PR was associated with increased diastolic HDFs along the diaphragm-to-RVOT direction, resulting in an RV deformation along the same direction and in a decrease of tricuspid annular plane systolic excursion (TAPSE).^40^ The identification of patients based on HDFs and shape variations described in the study may aid in determining the optimal timing of PVR.

## Future perspectives

HDF analysis is a promising tool in cardiovascular medicine as it plays a crucial role in regulating LV structure and function. Impaired intracardiac HDFs may predict adverse LV remodelling even earlier than traditional techniques, such as LVEF or strain echocardiography. HDFs were first assessable only through 4D-flow MRI or echo-PIV, which are expensive and time-consuming methods requiring highly specialized centres. However, recent advancements have made HDF analysis more accessible through cine CMR and TTE. This allows for a broader clinical application of HDF analysis, making it feasible for routine use in a wider range of healthcare settings.

**Figure 8:**
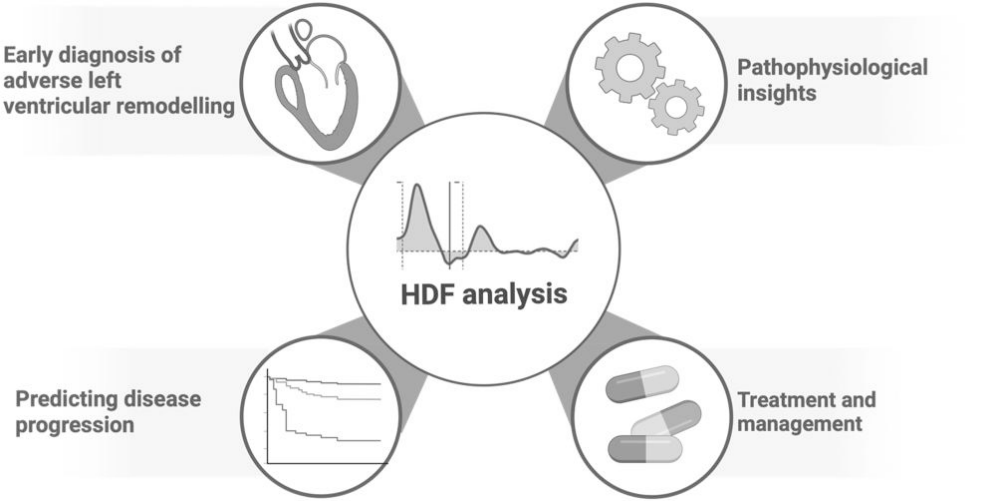
Potential applications of HDF analysis.

HDFs may offer insight into the intricate dynamics of cardiac function and remodelling, making them a valuable tool in both diagnostic and therapeutic contexts (*Figure 8*). This may also translate into a deeper understanding of the pathophysiology of various cardiovascular diseases such as HF, hypertensive, or valvular heart diseases. Finally, HDF analysis could offer invaluable insights into predicting disease progression and response to treatment beyond traditional clinical and echocardiographic parameters.

Although promising, the HDF analysis technique is not without limitations (*Table 3*). Available studies were performed predominantly on small sample sizes and in a retrospective manner, so larger and prospective studies are needed to draw definitive conclusions. There is also a need for standardization in measurement techniques and nomenclature to ensure consistency and comparability. Regarding the technique itself, HDF analysis exploits endocardial edge tracking technology, both in the case of echocardiography and CMR. Therefore, it is not currently applicable to patients with irregular rhythms, such as AF, in the presence of conditions that might alter the haemodynamic status or for cardiac diseases affecting merely epi-mesocardial myocardial layers. CMR, although more expensive and time-consuming, has the potential to overcome the constraints of echocardiography due to inadequate acoustic windows. Finally, being a time-consuming method may hinder its application in daily clinical practice and confine HDF analysis to research purposes at the moment.

**Table 3:** Main pitfalls in HDF analysis

Pitfall	Description
Relatively high cost	4D-flow MRI and advanced echocardiographic techniques are expensive and require specialized equipment and software
Time-consuming	Data acquisition and analysis can be time-intensive, with post-acquisition data analysis taking approximately 30 min per patient
Limited availability	Highly specialized centres with access to advanced imaging technology and expertise are required
Image quality dependency	Accurate HDF assessment requires high-quality imaging, which can be challenging in patients with poor acoustic windows or movement artefacts
Irregular rhythms	HDF analysis is not currently applicable to patients with irregular heart rhythms, such as atrial fibrillation.
Endocardial edge tracking	The technique relies on endocardial edge tracking, which may not be suitable for diseases affecting epi-mesocardial layers
Standardization issues	Lack of standardized measurement techniques and nomenclature can lead to inconsistencies and difficulties in comparing results across studies
Applicability in daily practice	The method’s current time consumption and complexity may hinder its routine use in daily clinical practice, confining it mostly to research
Limited patient data	Most studies have been conducted on small sample sizes and in a retrospective manner, necessitating larger, prospective studies for validation

Looking ahead, the HDF technique requires faster data analysis, potentially in real-time, to be effectively applied in everyday clinical practice. Future research should also focus on expanding the scope of HDF applications, exploring their potential in a wider range of cardiovascular conditions, including valvular diseases and cardiomyopathies. This implies not only enhancing our understanding of disease mechanisms but also improving patient management strategies, so that the integration of HDF analysis in clinical practice could pave the way for more personalized and effective treatments in cardiovascular medicine.

## Data Availability

No new data were generated or analysed in support of this research.

## Lead author biography

Dr. Aimo is a cardiologist at the Fondazione Toscana Gabriele Monasterio (a section of the Italian National Research Council) and a researcher at the Scuola Superiore Sant’Anna in Pisa, Italy. His research activity has mostly focused on heart failure and cardiomyopathies, particularly idiopathic dilated cardiomyopathy, and cardiac amyloidosis, but also cardiac involvement in mitochondrial disease or muscular dystrophies. His main fields of interest are the use of biomarkers or imaging findings as tools for diagnosis and management of heart failure and cardiomyopathies.
